# Health technology assessment of surgical robots: a mini-review of a rapidly evolving field

**DOI:** 10.3389/fpubh.2025.1672583

**Published:** 2025-10-30

**Authors:** Tiantian Zhang, Yunwu Zhong, Jia Zeng, Gordon G. Liu

**Affiliations:** 1 School of Pharmaceutical Sciences, Southern Medical University, Guangzhou, China; 2 College of Pharmacy/Southern Institute of Pharmacoeconomics and Health Technology Assessment, Jinan University, Guangzhou, China; 3 National School of Development, Peking University, Beijing, China

**Keywords:** surgical robots, health technology assessment, value-based healthcare, economic evaluation, challenges, real-world evidence

## Abstract

**Background:**

Surgical robots enhance precision and enable minimally invasive procedures but pose challenges for traditional Health Technology Assessment (HTA) due to high costs, organizational disruption, and rapid iteration.

**Core content:**

This review first reviews the development trajectory of surgical robots and their applications in modern surgery. It then elaborates on the fundamental definition and value dimensions of HTA, highlighting the IDEAL framework specifically designed for evaluating complex surgical innovations and its tailored recommendations for surgical robots. Then provides a detailed analysis of specific challenges encountered in HTA of surgical robots.

**Discussion:**

This review thoroughly examines two core controversies in current assessment paradigms: the debate over the roles of randomized controlled trials (RCTs) vs. real-world evidence (RWE) in the evidence hierarchy, and the paradigm tension between traditional cost-effectiveness analysis and the broader Value-Based Healthcare (VBHC) approach. Key identified research gaps include: the lack of standardized HTA frameworks accounting for medical device characteristics, insufficient long-term patient-centered outcomes and system-level impact data. Looking ahead, the assessment of surgical robots is evolving toward dynamic, iterative “living” HTA models, urgently requiring new paradigms to evaluate AI-integrated intelligent systems and ultimately striving toward a comprehensive assessment system integrating broad value dimensions with a global perspective.

**Conclusion:**

Static HTA methods, primarily designed for pharmaceuticals, are inadequate for complex, rapidly evolving platforms like surgical robots. Establishing a more dynamic, holistic, and standardized assessment paradigm is crucial to ensure safe, effective, and cost-efficient benefit for patients and society.

## 1 Introduction

Surgical robots evolved over decades, originating from Karel Capek's 1920 term “robot” (1) and Heinlein's 1942 vision of remote surgery (2). Military/space needs (DARPA/NASA) drove early “telesurgery” research for remote battlefield/space medical care, developing telepresence foundations (1). The PUMA 560 marked the first clinical use in 1985 (neurosurgery). The 1990s saw purpose-built systems like PROBOT, ROBODOC (first FDA-approved), and voice-controlled AESOP (1, 2). A paradigm shift occurred in the early 2000s with teleoperated master-slave systems, culminating in the da Vinci Surgical System receiving FDA clearance in 2000. Dominating the market with features like immersive 3D HD vision and EndoWrist^®^ instruments, da Vinci ushered in the robotic minimally invasive surgery era, demonstrating the evolution from single-function tools to sophisticated multi-functional platforms with escalating technical complexity and clinical utility (2).

Surgical robots now utilized broadly beyond initial urology/gynecology to nearly all laparoscopic specialties (e.g., cardiothoracic, colorectal), complicating evaluation. Adoption stems from significant advantages: for surgeons—enhanced precision (tremor filtration, motion scaling), superior 3D HD visualization, improved instrument maneuverability, and critical ergonomic benefits reducing fatigue/injury risk, potentially extending careers. For patients, this translates to key perioperative benefits: reduced pain/blood loss, shorter hospitalization, faster recovery, smaller scars, and lower complication risks (3, 4). These combined advantages constitute the compelling value proposition driving healthcare institutions' substantial investment.

As mentioned above, the core value proposition of surgical robots is compelling. However, it is met with a significant paradox: the immense cost and system-wide disruption associated with adopting surgical robots. The initial capital investment for a surgical robot can exceed $2 million, with substantial ongoing costs for maintenance, specialized training, and single-use instruments that can add thousands of dollars to each procedure (5). Given surgical robots' high costs/workflow disruption, Health Technology Assessment (HTA) is urgent for policymakers to evaluate efficacy/impact scientifically. However, traditional HTA frameworks face unprecedented challenges: unlike stable pharmaceuticals suited for static models, surgical robots are dynamic, evolving platforms. Their value depends on operator skills, software updates, adoption patterns, and hospital integration depth. This core challenge is a paradigm mismatch—applying static, single-indication assessment frameworks to dynamic, multi-indication platforms with high operator/context dependency. This underscores two research gaps: the current lack of standardized HTA frameworks specifically designed for advanced medical devices such as surgical robots and the dearth of long-term patient-centered outcomes and system-level impact data (6). Subsequent sections analyze this disjunction, introducing value dimensions of HTA/IDEAL frameworks, dissecting implementation challenges, and exploring controversies/future directions.

## 2 The landscape of health technology assessment

### 2.1 Foundational definition of HTA

To understand the challenges surgical robots pose to HTA, it is first necessary to establish a clear definition of HTA's purpose and scope. In 2020, the World Health Organization, in collaboration with several international organizations, provided the following definition and explanation for HTA: HTA evaluates various interventions for disease prevention, diagnosis, treatment, health promotion, and rehabilitation, including pharmaceuticals, biologics, medical devices, health materials, medical protocols, operational procedures, organizational management systems, logistics support systems, etc. It provides scientific information and decision-making basis for healthcare technology choices to decision-makers at all levels, including governments, health insurance companies, patients, and healthcare professionals (7). This formal context underscores HTA's role as a critical tool for promoting an equitable, efficient, and high-quality health system.

### 2.2 The evolving dimensions of value

At the core of any HTA is the assessment of “value,” a concept whose definition has become increasingly sophisticated over time. Traditionally, HTA frameworks prioritized a narrow set of dimensions, focusing primarily on clinical effectiveness, safety, and cost-effectiveness (8). While these remain essential pillars of assessment, it has become increasingly clear that they fail to capture the full impact of complex health technologies.

Recognizing these limitations, the International Society for Pharmacoeconomics and Outcomes Research (ISPOR) introduced the “flower of value” for health technologies in 2017, which covers thirteen value elements and is widely recognized (9). As time has progressed, in 2023, the organization “No Patient Left Behind” in the United States released the “flower of value” for generalized cost-effectiveness analysis (GCEA), which originates from costs and effects and includes fifteen broader value elements in four categories. This is greatly beneficial for future health technology assessments (10). The mapping of the petals (i.e., value elements) of the old and new flowers is shown in Table 1.

**Table 1:** Mapping GCEA value petals to ISPOR value flower.

**Category**	**GCEA**	**ISPOR**
Uncertainty	Outcome uncertainty	Value of hope
		Reduction in uncertainty
	Disease risk reduction	Insurance value
	Value of knowing	-
Dynamics	Dynamic net health costs	Net costs
	Dynamic prevalence	-
	Societal discount rate	-
	Option value	Real option value
	Scientific spillover	Scientific spillovers
Beneficiary	Patient-centered health improvements	Quality-adjusted life years (QALYs) gained
		Severity of disease
	Equity	Equity
	Family and caregiver spillover	Productivity
Additional Value Elements	Community spillover	Fear of contagion
	Productivity	Productivity
	Adherence	Adherence improving factors
	Direct non-medical costs	Net costs

This evolution from the ISPOR Value Flower to the GCEA framework is not merely an academic update; it signifies a fundamental philosophical shift in HTA. The transition is from a technology-centric model, primarily concerned with the direct cost and clinical effect of an intervention, to a more system- and society-centric perspective that acknowledges the broader ripple effects of a technology's implementation. This shift is precisely what is required to properly evaluate surgical robots. The value of robotic surgery is often claimed to lie in areas that traditional models ignore, such as improved surgeon ergonomics (a long-term system benefit), enhanced organizational efficiency, and faster patient recovery that reduces the burden on family and caregivers (11, 12). The GCEA framework, by formally incorporating elements like “Family and Caregiver Spillover” and “Scientific Spillover,” provides the necessary vocabulary and analytical structure to begin capturing and quantifying these very value elements. The development of these more sophisticated frameworks is not happening in a vacuum; it is a direct response to the limitations of older models that were exposed by the unique challenges of assessing complex, multi-faceted interventions like surgical robots.

## 3 The IDEAL framework for surgical robotics

Unlike pharmaceuticals, which are standardized products that can be evaluated through double-blind, randomized controlled trials (RCTs), surgical innovations are complex interventions with several distinguishing characteristics (13). First, they are highly operator-dependent; the outcome of a procedure is inextricably linked to the skill, experience, and learning curve of the surgeon and the entire surgical team (14). Second, surgical innovations often undergo a period of rapid, iterative development, where the technique and associated devices are modified and refined in light of early clinical experience (15). Third, strong preferences among both surgeons and patients can make randomization ethically or practically difficult, a phenomenon known as a loss of equipoise (16). These unique features mean that applying the rigid, linear evaluation pathway designed for drugs is often inappropriate for surgery. To address this methodological impasse, the Idea, Development, Exploration, Assessment, and Long-term study (IDEAL) framework was established. It provides a structured, staged pathway for the evaluation of surgical innovations, paralleling the phased approach of drug trials but tailored specifically to the unique challenges of surgery.

Recognizing that surgical robots represent one of the most complex and disruptive classes of surgical innovation, the IDEAL Collaboration convened an international group of experts to develop specific guidance for their evaluation. The resulting consensus statement, “The IDEAL Framework for Surgical Robotics,” was published in Nature Medicine in 2024 and provides a comprehensive roadmap for assessing these technologies throughout their lifecycle (17). This framework represents a critical inflection point, moving the HTA conversation from a reactive critique of existing evidence to a proactive strategy for generating the right evidence at the right time. The framework provides evaluation recommendations for the development, comparative research, and clinical monitoring of surgical robots from the perspectives of device developers, clinical doctors, patients, and the broader healthcare system (17). The three stages are as follows: IDEAL stages 0, 1, and 2a, which involve early clinical research on the safety and feasibility of the new concept of surgical robots; IDEAL stages 2b and 3, which involve larger-scale studies on the effectiveness of robotic interventions and comparing them with the current best practices; and IDEAL stage 4, which focuses on long-term monitoring of performance in real-world settings when robots are widely adopted.

By codifying this multi-stage, multi-perspective approach, the IDEAL framework provides a procedural solution to the methodological impasse that has long plagued the HTA of surgical robotics. It shifts the focus from criticizing the lack of perfect evidence at a single point in time to proactively generating a portfolio of appropriate, stage-specific evidence throughout the technology's entire lifecycle.

## 4 Considerations and suggestions for HTA of surgical robots

While the IDEAL framework provides a strategic roadmap, the practical implementation of HTA for surgical robots is fraught with specific methodological and logistical challenges. These issues cut across the domains of evidence synthesis, stakeholder engagement, economic analysis, and organizational integration, and must be carefully addressed to produce meaningful and reliable assessments.

### 4.1 Inclusion and exclusion of evidence

Clinical research on surgical robots is usually limited, resulting in a scarcity of literature. Additionally, due to the lack of appropriate controls, randomization, and blinding, the quality and reliability of evidence generated in clinical research may be compromised. Moreover, these studies are often small in scale, which may limit their generalizability when evaluating surgical interventions (18). Therefore, it is worth considering the inclusion of other types of evidence such as case reports, cohort studies, case-control studies, and real-world studies, while paying attention to the quality of the evidence.

### 4.2 Perspectives of patients and surgeons

Focusing only on rigid clinical outcomes in the health technology assessment of surgical robots may overlook factors that provide information for health policy decision-making, such as patient benefits, or ergonomic benefits for surgeons. Therefore, it is necessary to consider results that reflect the perspectives of patients and surgeons, such as patient preferences and satisfaction, as well as the comfort and efficiency of surgeons during the surgical process.

### 4.3 Learning curve effects

As the number of practice sessions increases, the performance of surgeons in using surgical robots gradually improves, leading to different health outcomes, which further affects the associated costs and introduces uncertainty in the assessment. Therefore, when conducting health technology assessment for surgical robots, the differences in clinical abilities among surgeons should be considered, and the learning curve effect should be corrected whenever possible (19). This requires using data that is suitable for the surgical volume and the experience with surgical robots in the specific study setting when selecting evidence of clinical effectiveness. It is also necessary to analyze the changes in clinical evidence related to surgical robots over time to observe if their performance is stable. Sensitivity analysis should be used to explore the impact of corresponding changes in performance. For quantifying the learning curve, a three-stage method proposed by the European network for Health Technology Assessment can be referred to.

### 4.4 Allocation of costs

Surgical robots themselves do not directly perform the intervention but need to be incorporated into one or more surgeries. However, in some health technology assessments, allocating the entire capital cost of the surgical robot system to a single surgery volume may result in biased outcomes. A more reasonable approach is to allocate the cost across all surgeries performed with the robot, calculating the cost based on the actual number of surgeries performed by the surgical robot. Only in this way can the current utilization of surgical robots in covering various patient surgeries by hospitals or healthcare systems be more comprehensively reflected.

### 4.5 Analysis methods

Currently, many economic evaluations of surgical robots only consider the costs compared to traditional surgical methods, making the assessment results not comprehensive and having limited reference value. The value-based healthcare (VBHC) approach should be used, which reflects the pursuit of the best clinical outcomes with the same or lower costs, maximizing the value obtained from healthcare services rather than simply comparing costs.

### 4.6 Appropriate time horizons

When evaluating the surgical outcomes of surgical robots, it is necessary to consider long-term impacts and set a reasonable time horizon. This includes the long-term effects on patient quality of life and economic costs, as well as the potential long-term effects on the wellbeing of surgeons. These impacts may not immediately manifest and require long-term tracking and research to fully assess. For example, certain clinical outcomes (such as recurrence) may only become apparent years after the surgery. Once these clinical outcomes occur, they can have a long-lasting impact on patient quality of life and economic costs, potentially even lasting a lifetime.

### 4.7 Organizational impact

Surgical robots can bring additional benefits to the entire organization of a hospital, which are often not considered in health technology assessments. These benefits include improved hospital operational efficiency, facilitation of data analysis, and remote surgery capabilities. Therefore, the value of the entire robotic ecosystem should be taken into account. Additionally, the implementation of surgical robots often requires substantial organizational investments and adaptations, such as new infrastructure and the creation and supervision of multidisciplinary teams. If the cost of implementing surgical robots is borne by healthcare providers, the analysis should include the costs of setting up the robotic-assisted surgical platform and the expenses incurred for optimizing the use of the robot platform, such as training. The impact of these costs should be evaluated in sensitivity analyses (20). Furthermore, the proportion of open and laparoscopic surgeries that may be replaced by robot-assisted surgeries should also be considered.

### 4.8 Incremental innovation

Surgical robot devices and their technologies are constantly evolving, especially with the integration of artificial intelligence (AI). The introduction of new models or products can influence clinical outcomes and costs, rendering health technology assessments quickly outdated or rendering the research process itself ineffective (17). To address these issues, innovative and iterative evaluation strategies such as implementation trials can be employed (21). Additionally, the Bayesian approach, which integrates prior knowledge and continuously incorporates new information, is more applicable in these cases (18).

## 5 Discussion

Having delineated the specific challenges for surgical robot HTA in preceding sections, this segment elevates the discussion to macro-perspectives—probing deeper into the current intellectual divergence within the assessment field, critical research voids, and future trajectories. This transcends mere problem dissection to explore how new evaluative paradigms might co-evolve with this complex technology.

### 5.1 Core controversies in assessment methodologies

Two pivotal controversies currently fragment the surgical robotics assessment landscape, reflecting not merely methodological disagreements but fundamental philosophical conflicts regarding evidence standards and value definitions.

#### 5.1.1 Randomized controlled trials (RCTs) vs. real-world evidence (RWE)

Surgical robots evaluation challenges randomized controlled trials (RCTs) due to ethical/practical barriers (no blinding, preference hindering randomization), the confounding surgeon learning curve affecting generalizability, and high cost/slow pace mismatched with rapid tech evolution (6, 22). Despite RCTs like ROLARR showing no significant advantage over laparoscopy, adoption rises, suggesting drivers beyond RCT metrics (23). Real-world evidence (RWE) emerges as a pragmatic complement/alternative, assessing broader populations and long-term effectiveness under real conditions, aligning with IDEAL framework monitoring (17, 24). However, RWE carries risks of bias, confounding, and variable data quality, potentially enabling low-evidence diffusion (14, 25). Framing RCTs/RWE as opposing is a false dichotomy; the solution requires a hybrid framework integrating both—leveraging RWE for targeted RCT design and using RCTs to validate RWE.

#### 5.1.2 Tension between cost-effectiveness analysis and holistic value-based healthcare (VBHC)

High robotic surgery costs spark debate between narrow cost-effectiveness analysis (CEA) and broader Value-Based Healthcare (VBHC). Traditional CEA finds robotics cost-ineffective due to high capital/recurring costs vs. cheaper alternatives. Conversely, VBHC defines value as “health outcomes per dollar across the full care cycle,” incorporating downstream savings (reduced complications, shorter stays) plus unquantified benefits: improved surgeon ergonomics, hospital organizational gains, and patient-centered outcomes (e.g., faster recovery, lower opioid use) (11, 12). This shift focuses from “cost per procedure” to “net long-term system value,” reflected in evolving frameworks like ISPOR's Value Flower/Generalized CEA, which formally incorporate elements like “value of hope” and “family and caregiver spillover.”

### 5.2 Current research gaps in HTA for surgical robotics

Despite growing discourse, critical knowledge and methodological gaps persist in the HTA of surgical robotics, demanding urgent attention from future research.

#### 5.2.1 The need for standardized device-specific assessment frameworks

The fundamental gap is the absence of a globally accepted, standardized HTA framework specifically designed for complex medical devices like surgical robots (6). Different HTA bodies employ varied methodologies, value dimensions, and key assumptions (e.g., annual procedure volume, depreciation period, time horizon), leading to significantly divergent, often contradictory or ambiguous conclusions even within the same country (6). This methodological chaos creates substantial uncertainty for manufacturers, hospital administrators, and policymakers, hindering the formation of a coherent evidence base. Rooted in this issue is the current system's attempt to forcibly apply a relatively static evaluation model designed for pharmaceuticals onto a multi-indication, operator-dependent, and continuously evolving device platform (15).

#### 5.2.2 Scarcity of longitudinal, patient-centered, and system-level outcome data

The vast majority of current studies focus on short-term perioperative clinical outcomes. A severe evidence gap exists regarding the long-term impacts of robotic surgery. This includes tracking critical oncological outcomes (e.g., long-term recurrence and survival rates), patients' long-term functional recovery status, and patient-reported outcomes throughout the disease course. Furthermore, rigorous quantification of the macro-level impact on the entire healthcare system is scarce. Examples include the effect of robotics on prolonging surgeons' careers, transforming overall operating room workflow efficiency, and its profound implications for hospital workforce allocation and training systems. In the absence of this longitudinal and system-level data, HTA potentially yields partial conclusions that overestimate or underestimate the technology's true value.

### 5.3 Future directions for surgical robotics and their evaluation

Synthesizing the preceding analysis, surgical robotics and their evaluation systems are evolving along several trajectories to address identified challenges and research gaps.

#### 5.3.1 Toward “living” HTA

The traditional model of one-time, static HTA reporting is obsolete for technologies like surgical robots undergoing continuous software updates and hardware iterations. The future lies in establishing dynamic or “Living” HTA models (26). Such models represent not a final report, but an ongoing evaluation process. They continuously integrate emerging evidence (especially RWE from large registry studies), enabling periodic updates to clinical practice recommendations and reimbursement policies. This approach acknowledges that the evidence base for any medical device technology evolves throughout its lifecycle (27). Germany's “fast-track” approval pathway for Digital Health Applications (DiGA), which allows provisional reimbursement while definitive evidence is gathered, provides a highly relevant real-world exemplar for this dynamic assessment paradigm (26).

#### 5.3.2 Developing novel evaluation frameworks for intelligent and autonomous systems

The integration of artificial intelligence (AI) will herald the next revolution in surgical robotics. It will transform robots from precise, remote-controlled instruments into “intelligent partners” capable of learning, providing decision support, and potentially performing partial autonomous actions in the future. This poses unprecedented evaluation challenges, as the subject is no longer a fixed device but a system potentially exhibiting a “black box” effect and capable of self-learning and adaptation. Future HTA frameworks must prepare accordingly. Recommendations from the IDEAL framework offer a starting point: independently evaluate the AI module first (e.g., via *in silico* simulation), followed by evaluation of the AI-integrated robotic system in clinical settings (17). Novel frameworks specific to digital health (e.g., Digi-HTA) are also being developed (28). Conversely, AI technology itself can empower the HTA process, for instance, by using natural language processing and machine learning to accelerate literature screening, data synthesis, and economic model building, thereby enhancing evaluation efficiency and depth (29). A core future task is developing methodologies capable of effectively validating, monitoring, and evaluating the safety, efficacy, and value of such dynamically learning systems.

#### 5.3.3 Integrating the full value spectrum and a global perspective

The future of surgical robotics HTA must ultimately become holistic, embracing both the full spectrum of value and addressing globalization impacts. At the practical level, this necessitates the widespread adoption and refinement of expanded value frameworks like GCEA (10), ensuring decisions are based on a comprehensive understanding of the technology's value rather than narrow cost considerations. At the policy level, global collaboration and effort are essential. International forums and cooperative mechanisms must be established to assess and mitigate the potential for expensive technology diffusion to exacerbate global health inequities. Recently, Italy's National Agency for Regional Health Services (AGENAS) initiated a nationwide HTA solicitation for robotic surgery systems, signaling a move toward more systematic assessment by national bodies (30). This trend needs to become a global consensus, driving the establishment of a more equitable, transparent, and sustainable global governance framework for surgical innovation.
