# Correction: Health technology assessment of surgical robots: a mini-review of a rapidly evolving field

**DOI:** 10.3389/fpubh.2025.1755969

**Published:** 2025-12-11

**Authors:** Tiantian Zhang, Yunwu Zhong, Jia Zeng, Gordon G. Liu

**Affiliations:** 1 School of Pharmaceutical Sciences, Southern Medical University, Guangzhou, China; 2 College of Pharmacy/Southern Institute of Pharmacoeconomics and Health Technology Assessment, Jinan University, Guangzhou, China; 3 National School of Development, Peking University, Beijing, China

**Keywords:** surgical robots, health technology assessment, value-based healthcare, economic evaluation, challenges, real-world evidence

Affiliation College of Pharmacy/Southern Institute of Pharmacoeconomics and Health Technology Assessment, Jinan University, Guangzhou, China was omitted from the paper. This affiliation has now been added for author Tiantian Zhang.

The original version of this article has been updated.

